# Supplementary figures and images for: The Impact of Population Demography and Selection on the Genetic Architecture of Complex Traits
Source: PLoS Genet. 2014 May 29;10(5):e1004379. doi: 10.1371/journal.pgen.1004379 (PMC4038606; doi:10.1371/journal.pgen.1004379)

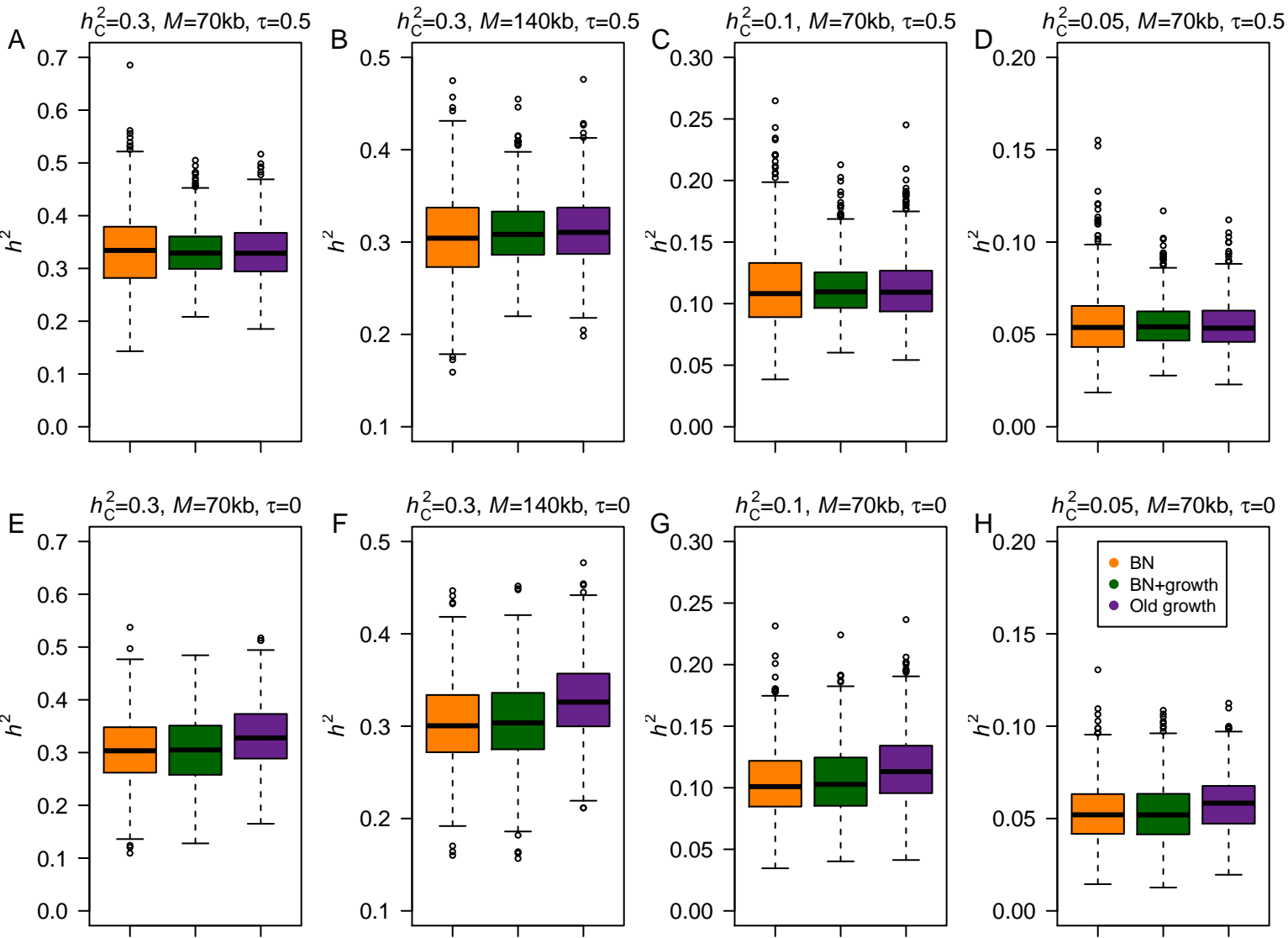

Supplement: Figure S1 — Population history has little effect on the narrow-sense heritability (h2) of a trait. (A–D) A SNP's effect on the trait is correlated with its effect on fitness (τ = 0.5). (E–H) A SNP's effect on the trait is independent of its effect on fitness (τ = 0). (A, E) and M = 70 kb. (B, F) and M = 140 kb. (C, G) and M = 70 kb. (D, H) and M = 70 kb. Narrow sense heritability was computed for each demographic model as . Here for all scenarios. , pi is the frequency of the i th SNP, and αi is the i th SNP's effect on the trait. (PDF) [file pgen.1004379.s001.pdf]

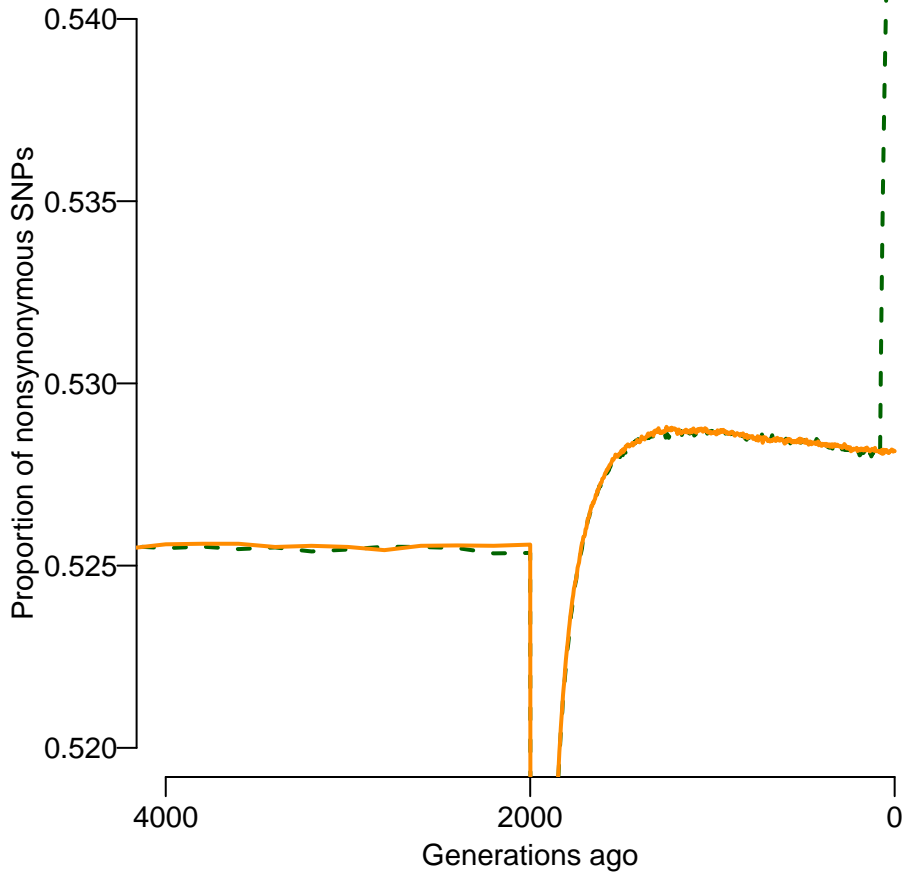

Supplement: Figure S2 — Proportion of nonsynonymous SNPs over time. Note that the proportion of nonsynonymous SNPs after the bottleneck (near 0 generations) is higher than that in the ancestral population (at time 4000 generations ago) for both the model with recent population growth (dashed green line) and the model without recent population growth (solid orange line). (PDF) [file pgen.1004379.s002.pdf]

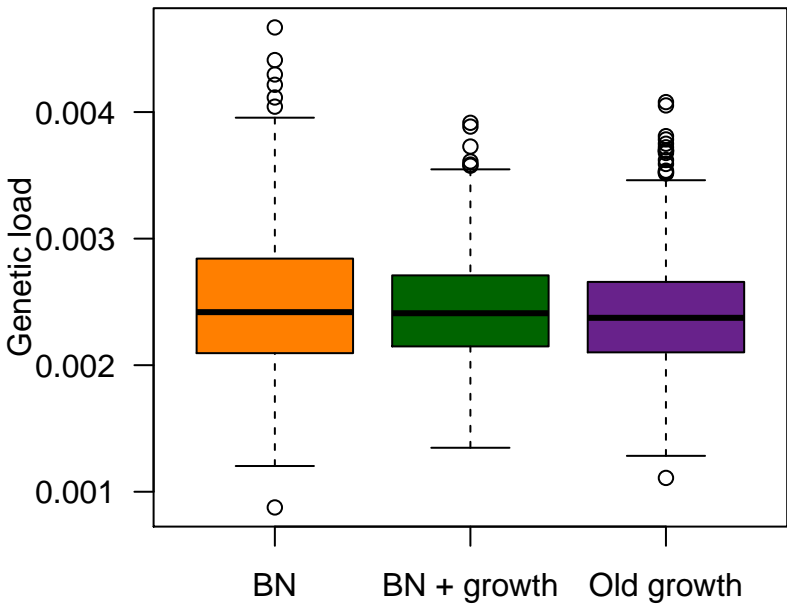

Supplement: Figure S3 — Population history has little effect on the genetic load. Genetic load was calculated for all SNPs segregating in a sample of 6000 individuals taken from each demographic history. (PDF) [file pgen.1004379.s003.pdf]

**A**  $h_C^2=0.3$ ,  $M=140\text{kb}$ ,  $\tau=0.5$ 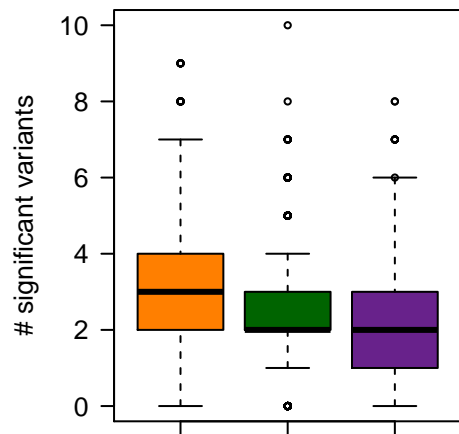**B**  $h_C^2=0.1$ ,  $M=70\text{kb}$ ,  $\tau=0.5$ 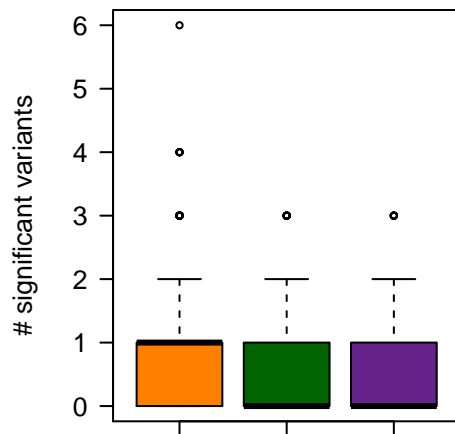**C**  $h_C^2=0.05$ ,  $M=70\text{kb}$ ,  $\tau=0.5$ 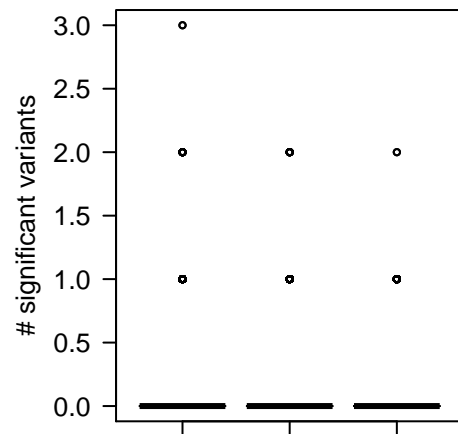**D**  $h_C^2=0.3$ ,  $M=140\text{kb}$ ,  $\tau=0$ 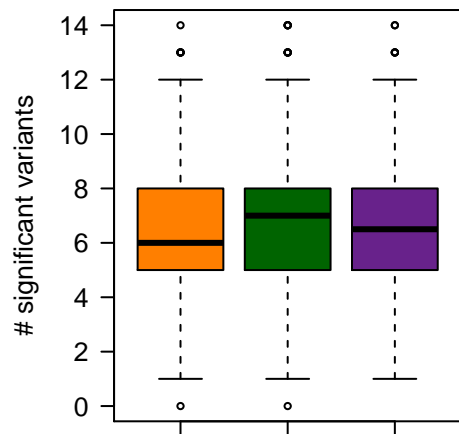**E**  $h_C^2=0.1$ ,  $M=70\text{kb}$ ,  $\tau=0$ 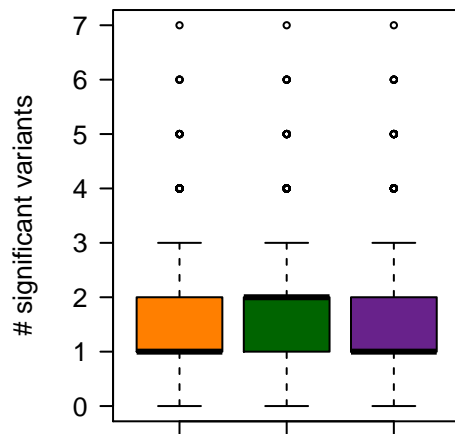**F**  $h_C^2=0.05$ ,  $M=70\text{kb}$ ,  $\tau=0$ 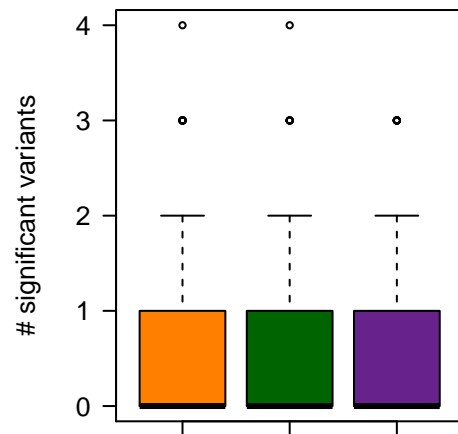

Supplement: Figure S4 — The number of causal SNPs with a significant P-value (<1×10^−5^) in the single-marker association test for additional models of the trait. Orange denotes the bottleneck demographic model (BN). Green denotes the bottleneck and recent growth model (BN+growth). Purple denotes the ancient growth model (Old growth). (A–C) A SNP's effect on the trait is correlated with its effect on fitness (τ = 0.5). (D–F) A SNP's effect on the trait is independent of its effect on fitness (τ = 0). (A, D) and M = 140 kb. (B, E) and M = 70 kb. (C, F) and M = 70 kb. (PDF) [file pgen.1004379.s004.pdf]

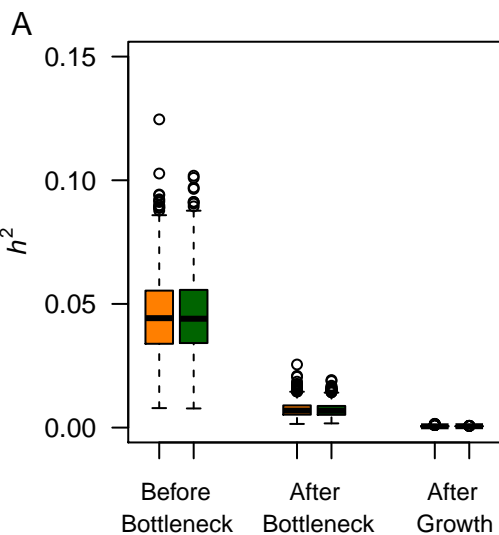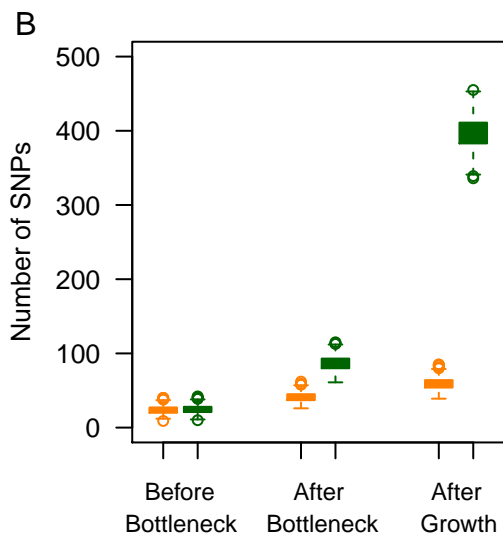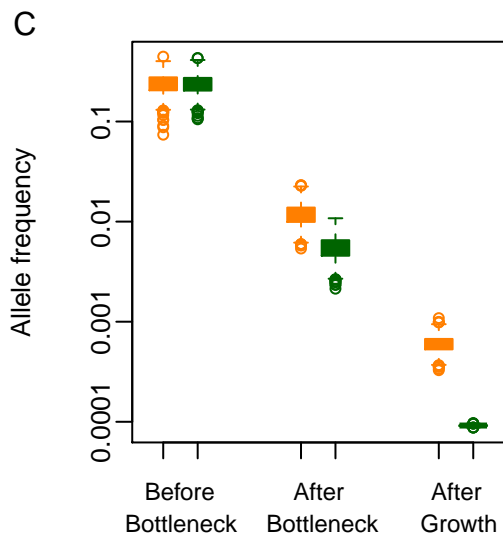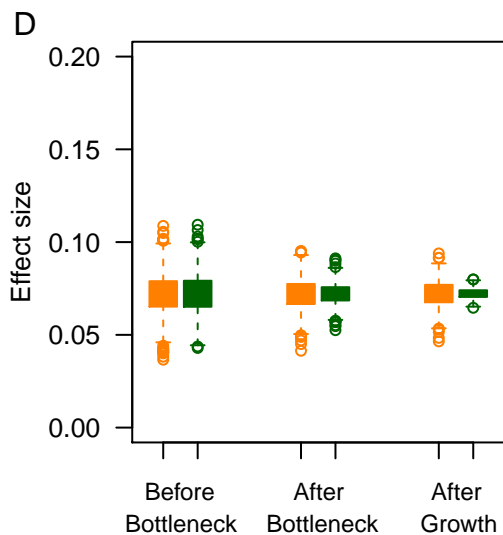

Supplement: Figure S5 — Effect of recent population growth on the heritability attributable to mutations of different ages when τ = 0. Orange boxes denote the bottlenecked population that did not recently expand (BN). Green boxes denote a population that expanded 80 generations ago (BN+growth). “Before bottleneck” refers to mutations that arose more than 1960 generations ago (before or during the bottleneck). “After bottleneck” refers to mutations that arose after the population recovered from the bottleneck, but earlier than 80 generations ago. “After growth” refers to mutations that arose within the last 80 generations (after the population expanded). (A) Heritability attributed to mutations of different ages. Note that recent population growth does not affect the median heritability attributable to mutations of different ages. (B) Number of SNPs segregating in the present-day that arose during the different time intervals. (C) Mean allele frequency of SNPs that are segregating in the present-day that arose during the different time intervals. (D) Mean effect size of SNPs that are segregating in the present-day that arose during the different time intervals. Here and M = 70 kb. (PDF) [file pgen.1004379.s005.pdf]

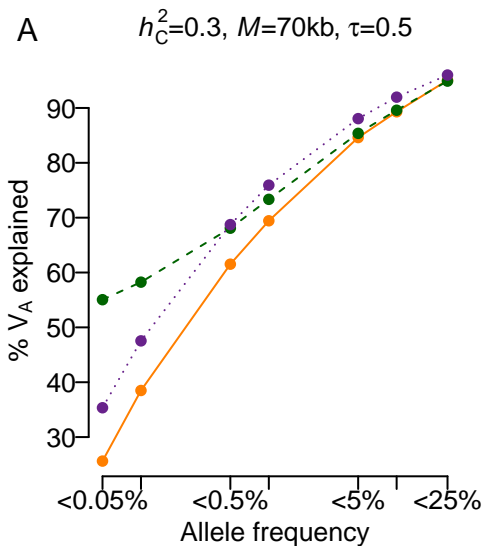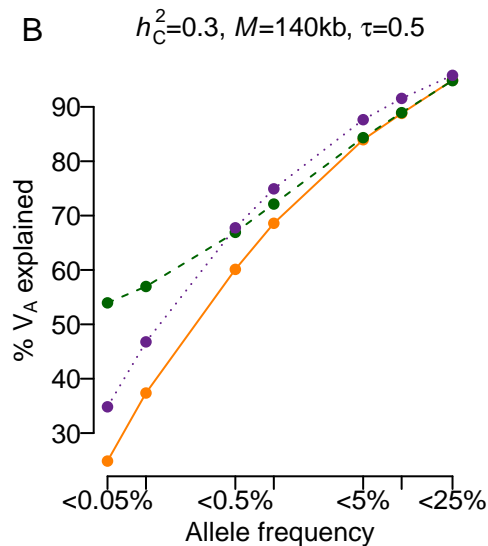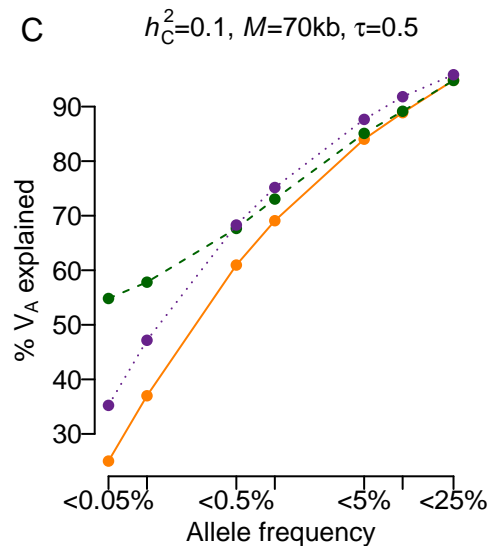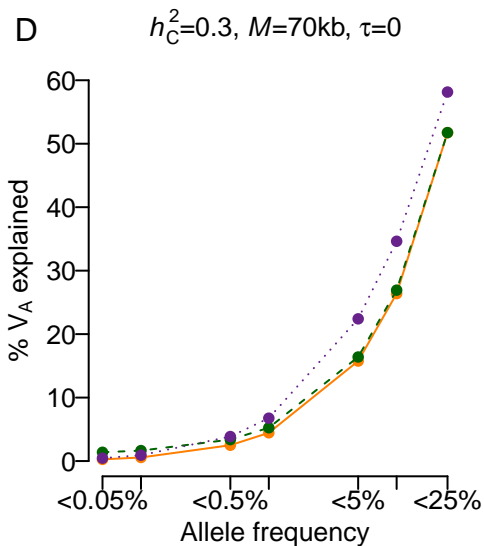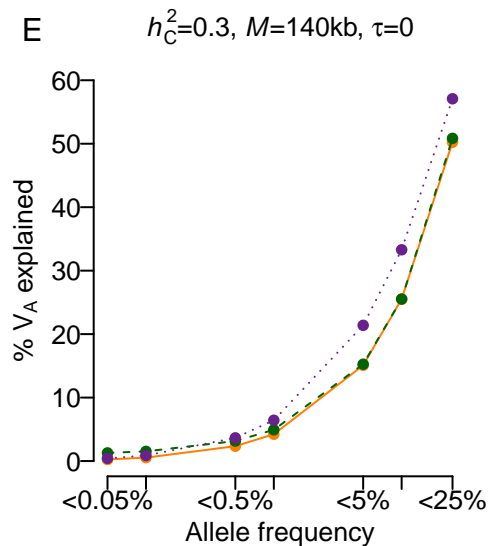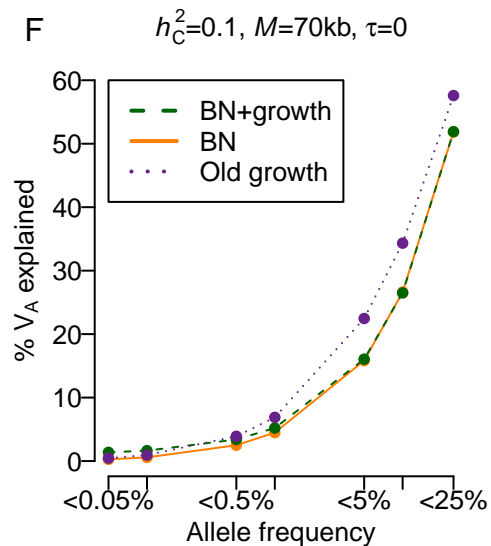

Supplement: Figure S6 — Cumulative distribution of the amount of the additive genetic variance of a trait (VA; y-axis) explained by SNPs segregating below a given frequency in the population (x-axis) for additional models of the trait. (A–C) A SNP's effect on the trait is correlated with its effect on fitness (τ = 0.5). Note that the population that experienced recent growth (green; BN+growth) has a higher proportion of VA accounted for by low-frequency SNPs (<0.1% frequency) than the populations that did not recently expand (orange and purple; BN and Old growth). (D–F) A SNP's effect on the trait is independent of its effect on fitness (τ = 0). Note that less of VA is accounted for by low-frequency variants than when the trait is correlated with fitness (A). (PDF) [file pgen.1004379.s006.pdf]

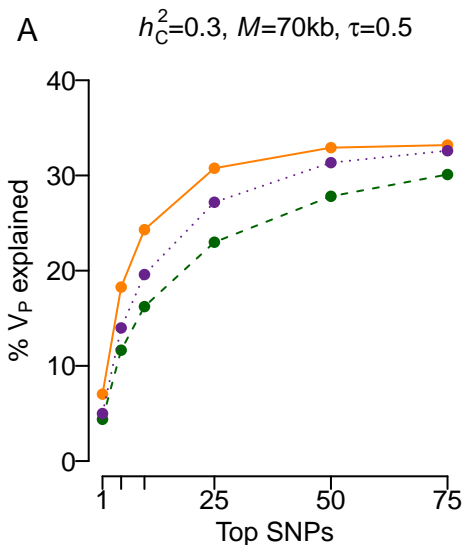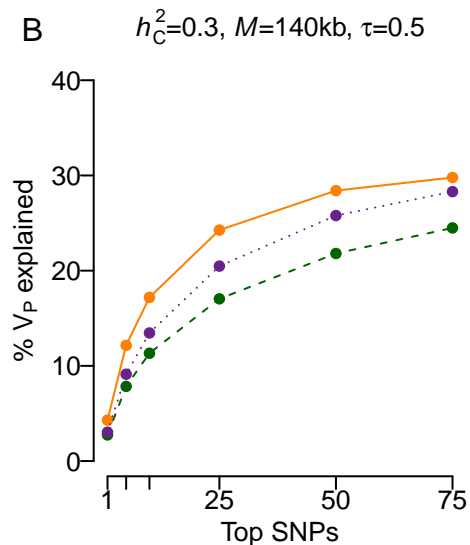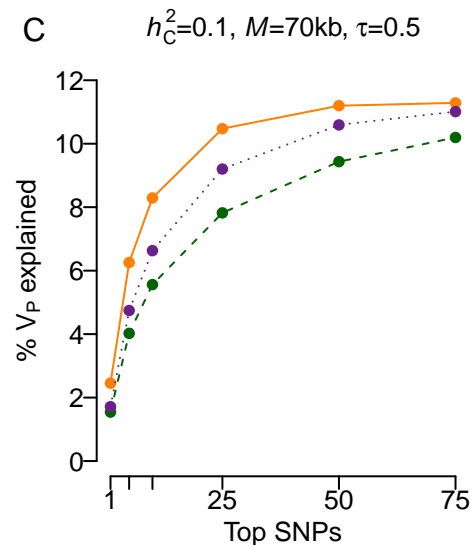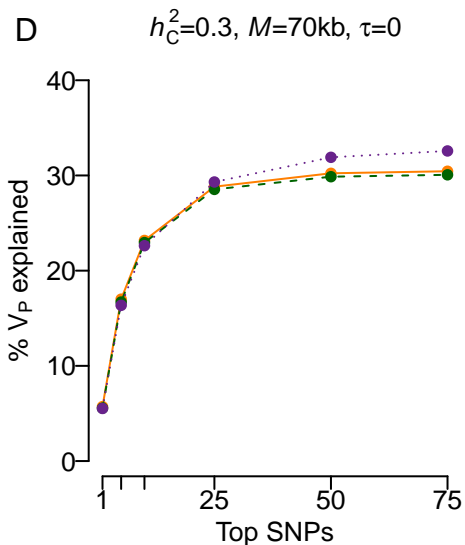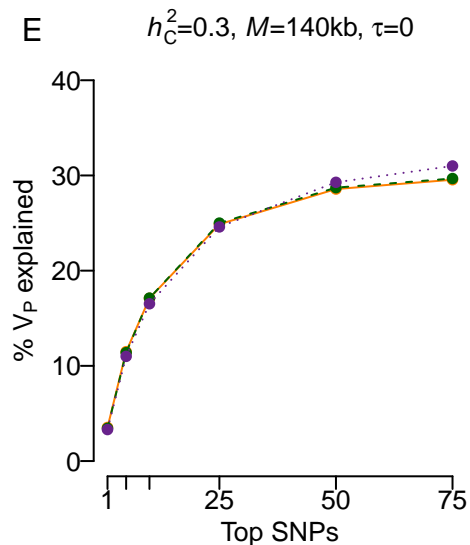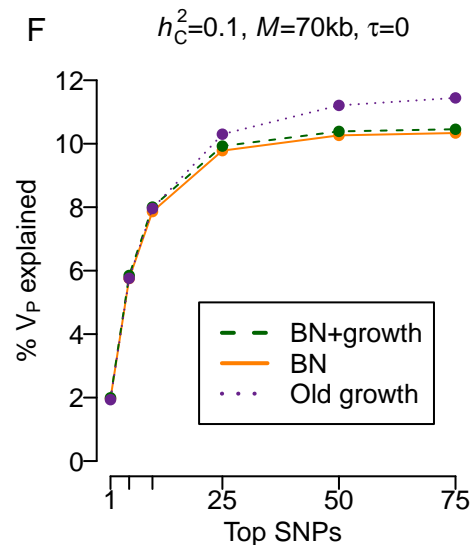

Supplement: Figure S7 — Cumulative distribution of the amount of the phenotypic variance of a trait (VP; y-axis) explained by the SNPs that explain the most variance (x-axis) for additional models of the trait. (A–C) A SNP's effect on the trait is correlated with its effect on fitness (τ = 0.5). Note that the top SNPs account for less of the phenotypic variance in the population that experienced recent growth (green; BN+growth) than in the populations that did not recently expand (orange and purple; BN and Old growth). (D–F) A SNP's effect on the trait is independent of its effect on fitness (τ = 0). (PDF) [file pgen.1004379.s007.pdf]

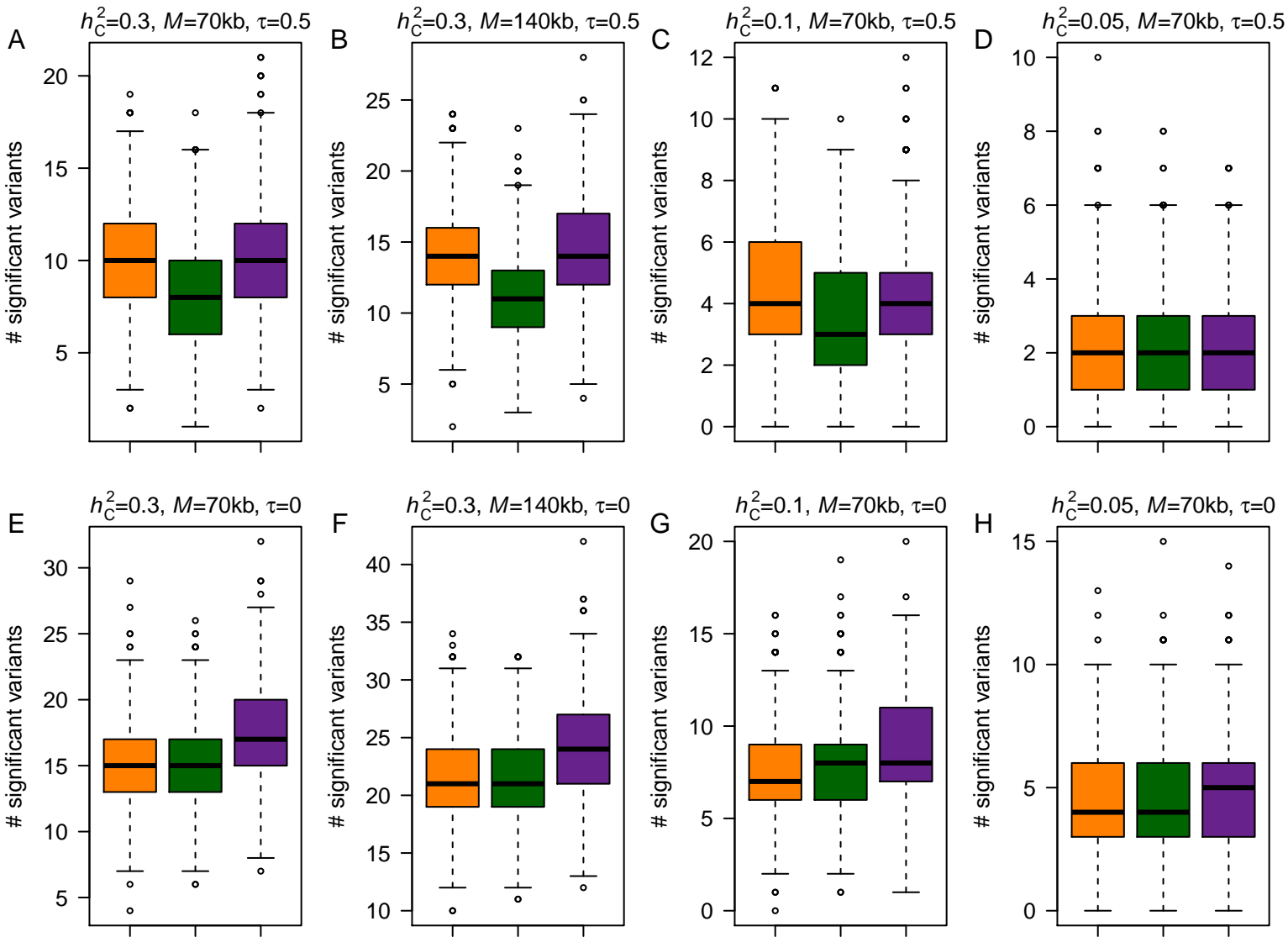

Supplement: Figure S8 — The number of causal SNPs with a P-value <1×10^−2^ in the single-marker association test for different models of population history and the trait. Orange denotes the bottleneck demographic model (BN). Green denotes the bottleneck and recent growth model (BN+growth). Purple denotes the ancient growth model (Old growth). (A–D) A SNP's effect on the trait is correlated with its effect on fitness (τ = 0.5). (E–H) A SNP's effect on the trait is independent of its effect on fitness (τ = 0). (A, E) and M = 70 kb. (B, F) and M = 140 kb. (C, G) and M = 70 kb. (D, H) and M = 70 kb. (PDF) [file pgen.1004379.s008.pdf]

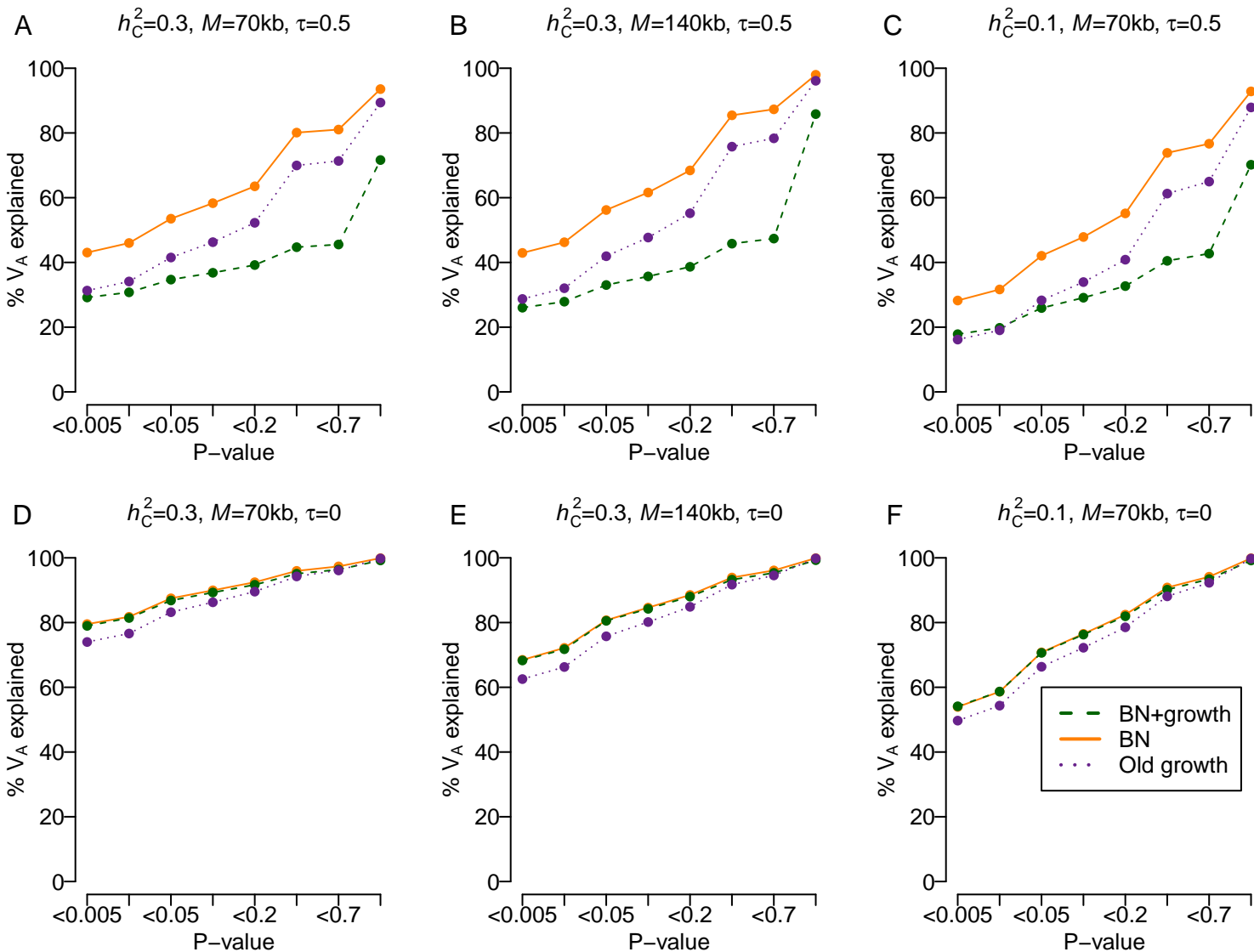

Supplement: Figure S9 — Cumulative distribution of the amount of the additive genetic variance of a trait (VA; y-axis) explained by SNPs with a single-marker association test P-value less than a given threshold (x-axis) for additional models of the trait. (A–C) A SNP's effect on the trait is correlated with its effect on fitness (τ = 0.5). Note that the population that experienced recent growth (green line; BN+growth) has a lower proportion of VA accounted for by SNPs at any P-value threshold than the populations that did not recently expand (orange and purple lines; BN and Old growth). (D–F) A SNP's effect on the trait is independent of its effect on fitness (τ = 0). Note that the SNPs with low P-values (<0.05) account for most of the VA regardless of the demographic history of the population. (A, D) and M = 70 kb. (B, E) and M = 140 kb. (C, F) and M = 70 kb. (PDF) [file pgen.1004379.s009.pdf]

A

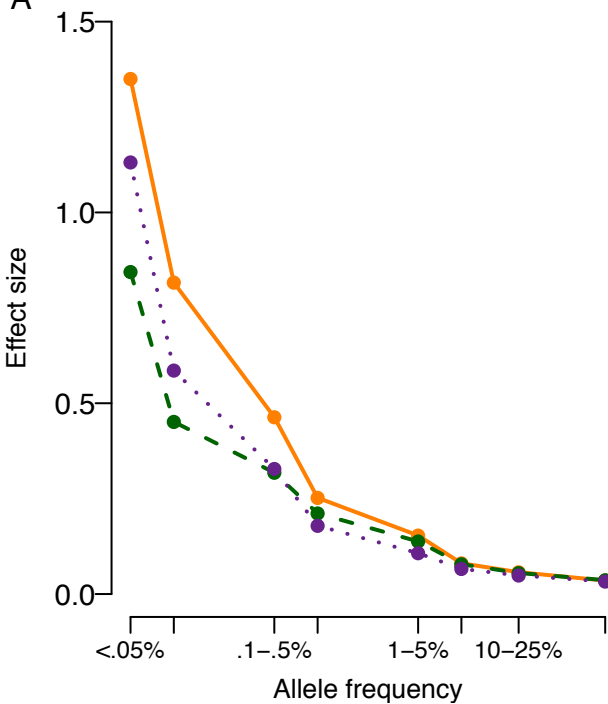

B

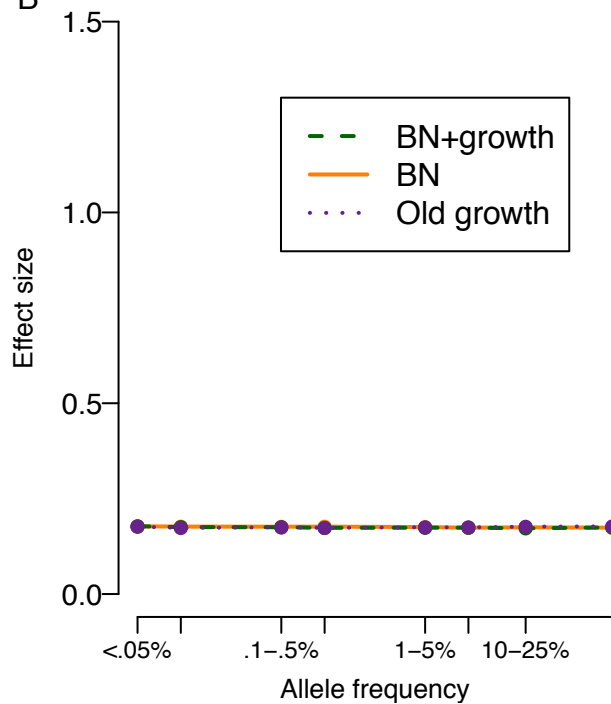

C

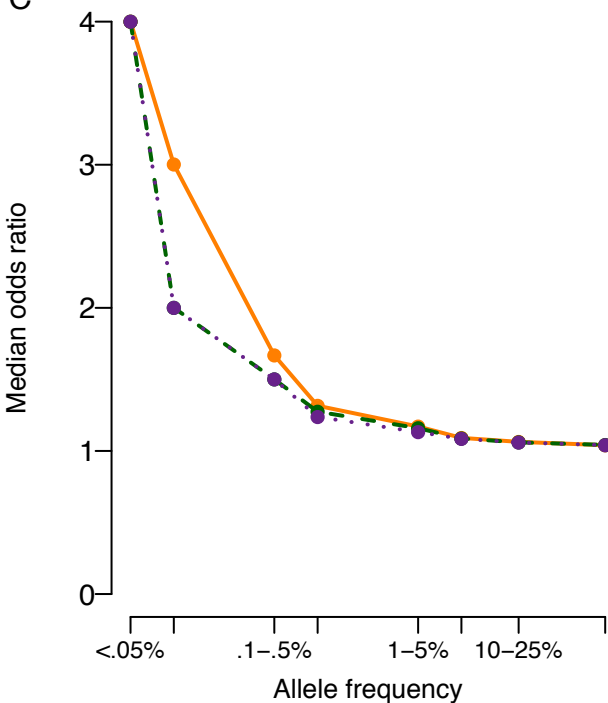

D

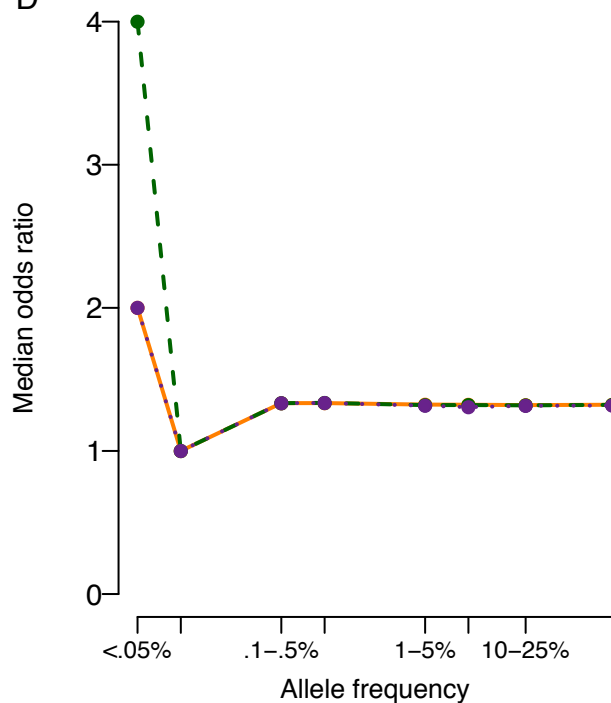

Supplement: Figure S10 — The relationship between a mutation's effect on the trait and its allele frequency. Statistics were calculated for each simulation replicate and then averaged over the 1000 simulation replicates. (A, C) A SNP's effect on the trait is correlated with its effect on fitness (τ = 0.5). (B, D) A SNP's effect on the trait is independent of its effect on fitness (τ = 0). (A–B) Average effect size (on the liability scale) for SNPs having the allele frequency (in the population) specified on the x-axis. (C–D) Median odds ratios (ORs) computed from a sample of 1000 cases and controls across all SNPs in a simulation replicate having the allele frequency (in the population) specified on the x-axis. Note, median ORs equal to infinity (due to many case-only variants) were set to 4 for plotting purposes. Here and M = 70 kb. (PDF) [file pgen.1004379.s010.pdf]
