# Supplementary material for: The Impact of Population Demography and Selection on the Genetic Architecture of Complex Traits
Source: PLoS Genet. 2014 May 29;10(5):e1004379. doi: 10.1371/journal.pgen.1004379 (PMC4038606; doi:10.1371/journal.pgen.1004379)
Supplement: Table S1 — Values of the constant C used to generate the desired heritability for different values of and M. (DOCX) [file pgen.1004379.s011.docx]

Table S1: Values of the constant *C* used to generate the desired heritability for different values of $h_{C}^{2}$ and *M*.

| τ | $h_{C}^{2}$ = 0.3; *M* = 70 kb | $h_{C}^{2}$ = 0.3; *M* = 140 kb | $h_{C}^{2}$= 0.1; *M* = 70 kb | $h_{C}^{2}$= 0.05; *M* = 70 kb |
| --- | --- | --- | --- | --- |
| 0 | 0.03058 | 0.014891 | 0.0104 | 0.005219 |
| 0.5 | 60 | 26.69925 | 19.24 | 9.435 |

τ denotes the relationship between a mutation’s effect on fitness and the trait. $h_{C}^{2}$ refers to the heritability that the simulation was calibrated to in a constant size population. Note, the same value of *C* was used under all models of population history (see Methods).
