# Supplementary material for: The Impact of Population Demography and Selection on the Genetic Architecture of Complex Traits
Source: PLoS Genet. 2014 May 29;10(5):e1004379. doi: 10.1371/journal.pgen.1004379 (PMC4038606; doi:10.1371/journal.pgen.1004379)
Supplement: Table S2 — Average percentage of the phenotype variance (VP) explained by the top 50 SNPs that explain the most variance under different models of population history, and M. (DOCX) [file pgen.1004379.s012.docx]

| τ | Population | **$h_{C}^{2}$** = 0.3; *M* = 70 kb | **$h_{C}^{2}$**= 0.3; *M* = 140 kb | **$h_{C}^{2}$** = 0.1; *M* = 70 kb | **$h_{C}^{2}$**= 0.05; *M* = 70 kb |
| --- | --- | --- | --- | --- | --- |
| 0 | BN+growth | 29.9 | 28.7 | 10.3 | 5.3 |
|  | BN | 30.2 | 28.6 | 10.3 | 5.3 |
|  | Old growth | 31.9 | 29.3 | 11.2 | 5.6 |
|  |  |  |  |  |  |
| 0.5 | BN+growth | 27.8 | 21.8 | 9.4 | 4.7 |
|  | BN | 32.9 | 28.4 | 11.2 | 5.5 |
|  | Old growth | 31.3 | 25.6 | 10.6 | 5.2 |

Table S2: Average percentage of the phenotype variance (*V_P_*) explained by the top 50 SNPs that explain the most variance under different models of population history, **$h_{C}^{2}$** and *M*.

τ denotes the relationship between a mutation’s effect on fitness and the trait. **$h_{C}^{2}$** refers to the heritability that the simulation was calibrated to in a constant size population. For this reason, some of the observed averages of *V_P_* may be greater than ****listed in the column heading. *M* refers to the mutational target size (see Methods). Note that certain models predict that the top 50 SNPs, in aggregate, explain <10% of the *V_P_*.
