# Supplementary material for: The Impact of Population Demography and Selection on the Genetic Architecture of Complex Traits
Source: PLoS Genet. 2014 May 29;10(5):e1004379. doi: 10.1371/journal.pgen.1004379 (PMC4038606; doi:10.1371/journal.pgen.1004379)
Supplement: Table S3 — Average number of GWAS hits expected in samples of 1000 cases and 1000 controls under different models of population history, and M. (DOCX) [file pgen.1004379.s013.docx]

Table S3: Average number of GWAS hits expected in samples of 1,000 cases and 1,000 controls under different models of population history, **$h_{C}^{2}$** and *M*.

| τ | Population | **$h_{C}^{2}$**= 0.3; *M* = 70 kb | **$h_{C}^{2}$** = 0.3; *M* = 140 kb | **$h_{C}^{2}$** = 0.1; *M* = 70 kb | **$h_{C}^{2}$** = 0.05; *M* = 70 kb |
| --- | --- | --- | --- | --- | --- |
| 0 | BN+growth | 3.9 | 3.3 | 0.6 | 0.1 |
|  | BN | 4.0 | 3.2 | 0.6 | 0.1 |
|  | Old growth | 3.9 | 3.1 | 0.6 | 0.1 |
|  |  |  |  |  |  |
| 0.5 | BN+growth | 1.4 | 1.2 | 0.2 | 0.0 |
|  | BN | 1.7 | 1.7 | 0.2 | 0.0 |
|  | Old growth | 1.1 | 0.88 | 0.1 | 0.0 |

τ denotes the relationship between a mutation’s effect on fitness and the trait. **$h_{C}^{2}$** refers to the heritability that the simulation was calibrated to in a constant size population. A significance threshold of 5 x 10^-8^ was used.
