# Supplementary material for: The Impact of Population Demography and Selection on the Genetic Architecture of Complex Traits
Source: PLoS Genet. 2014 May 29;10(5):e1004379. doi: 10.1371/journal.pgen.1004379 (PMC4038606; doi:10.1371/journal.pgen.1004379)
Supplement: Text S1 — Additional results on recent growth and deleterious variation. (DOCX) [file pgen.1004379.s014.docx]

**Supplementary Text S1**

**Additional results on recent growth and deleterious variation**

*Looking into the future: re-attaining equilibrium*

Very recent population growth leads to an increase in the proportion of nonsynonymous SNPs in the population compared to a population that has not recently expanded. Thus, the recent population growth has pushed the population out of equilibrium. But, as natural selection eliminates the new deleterious mutations from the population, the proportion of nonsynonymous SNPs in the population and the average deleterious effect of a SNP will decrease. Eventually, assuming no further demographic changes, the proportion of nonsynonymous SNPs in the population will attain the new equilibrium value for the larger population size (**Fig. S1.1**).

**Fig. S1.1:** **Figure S5: Re-attaining the equilibrium value for the proportion of nonsynonymous SNPs after a 5-fold population expansion**. The population instantaneously expanded 5-fold at time 0. Solid curve shows how the proportion of nonsynonymous SNPs is expected to change over time. The dashed line denotes the proportion of nonsynonymous SNPs at equilibrium for the larger population size. After approximately 4*N_e_* (where *N_e_* is the size after the expansion) generations, the proportion of nonsynonymous SNPs in the expanded population reaches the equilibrium proportion for the larger population size.

How long will it take for human populations to reach this new equilibrium? By running the simulations with 5-fold growth for many generations into the future, it will take roughly 200,000 or 4*N_e_* generations for the proportion of nonsynonymous SNPs segregating in the population to decrease to the new equilibrium value for the larger population size (**Fig. S1.1**). This agrees well with the classic result that, conditional on fixation, a new neutral mutation takes roughly 4*N_e_* generations to become fixed in the population [1]. Thus, extrapolating to 100-fold growth, it would take roughly 4 million generations to reach the equilibrium proportion of nonsynonymous SNPs in the population.

*Effect of different growth rates*

I also examine the effect that different magnitudes of population growth have on the proportion of nonsynonymous SNPs in the population (**Fig. S1.2A**) and the average fitness effects of segregating deleterious variants (**Fig. S1.2B**). As expected, stronger recent population growth results in a higher proportion of nonsynonymous SNPs in the population (**Fig. S1.2A**). Interestingly, the difference between 10-fold growth and 100-fold growth appears rather slight compared to the difference between 0 and 5-fold growth. This result suggests that even a small amount of recent growth may be sufficient to affect patterns of weakly deleterious mutations. The picture for the average fitness effect of segregating deleterious variants is more complex (**Fig. S1.2B**). Here, as the magnitude of growth increases, the average segregating SNP becomes less deleterious. This effect can be explained by selection being more efficient in the larger population (as suggested in a recent paper by Gazave et al. [2]). Interestingly, the 80 generations since the expansion has been sufficient time for selection to have begun removing some of the most deleterious mutations (**Fig. S1.3).**

**Figure S1.2: Changes in genetic variation as a function of the magnitude of recent population growth.** In all cases, populations instantaneously expanded by the factor shown on the *x*-axis 80 generations ago. (**A**) Proportion of SNPs segregating in the sample that are nonsynonymous. (**B**) Absolute value of the average fitness effect of nonsynonymous SNPs segregating in the sample. Samples of 1000 chromosomes were taken at the simulation, reflecting the patterns in the present day. Results are averaged over 1000 simulation replicates.

**Figure S1.3: Average fitness effect of a nonsynonymous variant segregating in populations that have undergone various amounts of growth in the last 80 generations.** Note that at 50 generations ago, the average SNP was most deleterious in the populations that experienced the greatest expansion (100-fold and 50-fold). This is due to the increased input of new deleterious mutations as a result of the population expansion. However, in the present day (0 generations ago), the average SNP is least deleterious in the populations that expanded the most. This is due to the increased efficacy of purifying selection in a large population. Thus, many of the most deleterious mutations are quickly eliminated from the population.

**REFERNECES**

1.     Kimura M, Ohta T. (1969) The average number of generations until fixation of a mutant gene in a finite population. Genetics 61: 763-771.

2.     Gazave E, Chang D, Clark AG, Keinan A. (2013) Population growth inflates the per-individual number of deleterious mutations and reduces their mean effect. Genetics 195: 969-978.
