# Supplementary material for: The Impact of Population Demography and Selection on the Genetic Architecture of Complex Traits
Source: PLoS Genet. 2014 May 29;10(5):e1004379. doi: 10.1371/journal.pgen.1004379 (PMC4038606; doi:10.1371/journal.pgen.1004379)
Supplement: Text S2 — Additional results on the effect of demography on the power of association tests. (DOCX) [file pgen.1004379.s015.docx]

**Supplementary Text S2**

**Additional results on the effect of demography on the power of association tests**

All results described here were obtained using $h_{C}^{2}=0.3$, *M* = 70 kb, and a significance level of 1 x 10^-5^ for Fisher’s exact test.

*Power as a function of V_A_*

I examine the power to detect an association as a function of the amount of the additive genetic variance (*V_A_*) that a given SNP explains (**Fig. S2.1**). When a mutation’s effect on fitness is correlated with its effect on the trait (τ = 0.5), power to detect an association with a SNP that explains much of the *V_A_* (>5%) is higher in the population that recently expanded (BN+growth; green line in **Fig. S2.1A**) than in a population that did not (BN; orange line in **Fig. S2.1A**). This increase in power comes from the fact that those SNPs that explain a lot of *V_A_* in the recently expanded population tend to have smaller effect sizes than those SNPs in the population that did not recently expand (**Fig. S2.1C**). Because, under this model, the effect size of a mutation is correlated with the strength of selection acting against it (**Fig. S10**), those SNPs that have smaller effect sizes are also less deleterious and can reach higher frequency in the population. This pattern is demonstrated in **Fig. S2.1E**, which shows that the SNPs that each account for about 5% of the *V_A_* in the BN+growth population have a higher average frequency than those SNPs that explain a similar proportion of *V_A_* in the BN population. This increase in allele frequency results in an increase in power for the single-marker association test. An obvious question is why do the SNPs that explain >5% of the *V_A_* in the BN+growth population have smaller effect sizes than SNPs that explain similar amounts of *V_A_* in the BN population? This counter-intuitive pattern can be explained by noting that in order for a given SNP to explain >5% of the *V_A_*, it must be relatively common, or have a large effect, or both. In the BN population, there are low-frequency mutations of strong effect which are moderately deleterious that may explain >5% of the *V_A_*. In the BN+growth population, however, many of these low-frequency deleterious mutations with strong effects are eliminated from the population due to the increased efficacy of purifying selection in the large population. Those mutations that are left behind in the recently expanded population that can explain >5% of the *V_A_* will tend to be less deleterious, have smaller effect sizes (**Fig. S2.1C**), but have higher allele frequencies compared to the mutations that explain >5% of the *V_A_* in the BN population (**Fig. S2.1E**). The recent population growth also increases the number of new deleterious mutations with large effect sizes that enter the population. However, these mutations are so rare that they are unlikely to contribute >5% of the *V_A_*, and so they are not relevant for the present discussion.

When a mutation’s effect on fitness is not correlated with its effect on the trait (τ = 0), I find that demography has little effect on the power of the association test (**Fig. S2.1B)**. For all three models of population history, power to detect mutations that explain more of the *V_A_* is high. Further, the SNPs that tend to explain most of the *V_A_* tend to be at higher allele frequency (**Fig. S2.1D**) and have larger effect sizes than those mutations that explain less *V_A_* (**Fig. S2.1F**).

**Figure S2.1: Properties of single-marker association tests for causal variants in a study of 1000 cases and 1000 controls, binned by the additive genetic variance explained by the SNP (*V_A_*; *x*-axis). (A, C, E)** A SNP’s effect on the trait is correlated with its effect on fitness (τ = 0.5). (**B, D, F**) A SNP’s effect on the trait is independent of its effect on fitness (τ = 0). **(A-B)** Power. **(C-D)** Average effect size of the causal variant on the liability scale. **(E-F)** Average allele frequency of the causal variants.

*Power to detect the SNPs that explain the most V_A_*

I also investigate the power to detect the top SNPs that explain the most *V_A_* in each simulation replicate (**Fig. S2.2**). Similar trends to those discussed above apply here as well. For example, when τ = 0.5, the power to detect the SNP that explains the most *V_A_* in a given simulation replicate is 46% in the BN population (orange line in **Fig. S2.2A**). Power to detect the top SNP in the BN+growth population is considerably higher—above 70%. This is due to the top SNPs being at higher allele frequency in the expanded population than in the non-expanded population (**Fig S2.2E**), resulting in an increase in power. Further, the top SNPs are less likely to be private to cases in the recently expanded population than in the population that did not expand (**Fig. S2.2C**). This pattern supports the idea that the top SNPs that explain the most *V_A_* are at higher frequency in the expanded population. Finally, as noted above, when a mutation’s effect on fitness is not correlated with its effect on the trait (τ = 0), demography has little effect on the power of the association test or the frequencies of the causal variants (**Fig. S2.2B**, **Fig. S2.2D**, and **Fig. S2.2F**).

**Figure S2.2: Properties of single-marker association tests for causal variants in a study of 1000 cases and 1000 controls for the SNPs that explain the most *V_A_* per simulation replicate (*x*-axis).** Statistics were calculated for each simulation replicate and then averaged over the 1000 simulation replicates. **(A, C, E)** A SNP’s effect on the trait is correlated with its effect on fitness (τ = 0.5). (**B, D, F**) A SNP’s effect on the trait is independent of its effect on fitness (τ = 0). **(A-B)** Power. **(C-D)** Proportion of causal variants that were segregating in cases only. **(E-F)** Average allele frequency of the causal variants.

*Power as a function of allele frequency*

Next I examine the power of the single-marker association tests as a function of the allele frequency of the SNP (**Fig. S2.3**). When a mutation’s effect on fitness is correlated with its effect on the trait (τ = 0.5), I find that power is highest for SNPs with allele frequencies between 5-10% (**Fig. S2.3A**). Additionally, power is slightly higher in the population that did not expand as compared to that in the expanded population. Power is low for rare SNPs, as expected. Power is also low for very common SNPs (>10%) because, under this model, these SNPs also have the smallest effect sizes, which leads to a decrease in power. While recent population growth only has a subtle effect on the power of the association test when conditioning on allele frequency, it has a substantial effect on the number of rare causal variants. As expected, population growth increases the number of rare causal variants (**Fig. S2.3C**). These variants are difficult to detect via single-marker association tests. Similar to previous results, when a mutation’s effect on fitness is not correlated with its effect on the trait (τ = 0), I find that demography has little effect on the power of the association test, and that the power of the test is highest for common variants (allele frequency >10%; **Fig. S2.3B**). Under this model, effect sizes are not correlated with allele frequencies (**Fig. S2.3B**), and as such, common variants are just as likely to have large effects as are rare variants. Thus, power is highest to detect common variants. Again, however, recent population growth increases the number of rare causal variants in the population (**Fig. S2.3D**), many of which will be difficult to detect using single marker association tests.

**Figure S2.3: Properties of single-marker association tests for causal variants in a study of 1000 cases and 1000 controls, binned by the causal variant allele frequency (*x*-axis).** Statistics were calculated for each simulation replicate, and then were averaged over the 1000 simulation replicates. **(A, C)** A SNP’s effect on the trait is correlated with its effect on fitness (τ = 0.5). (**B, D**) A SNP’s effect on the trait is independent of its effect on fitness (τ = 0). **(A, B)** Power. **(C, D)** Number of casual variants at different allele frequencies. Note that recent growth (BN+growth) drastically increases the number of rare causal variants (dashed green lines).

*Power as a function of the odds ratio (OR)*

I also examine the power of the association test as a function of the estimated odds ratio (OR) computed from the case-control study (**Fig. S2.4**). When a mutation’s effect on fitness is correlated with its effect on the trait (τ = 0.5), power is highest for those SNPs with an OR close to 10 (**Fig. S2.4A**). Power decreases as the effect sizes decreases. Also, there is essentially no difference in power across the different models of population history. Many SNPs were present only in cases. An OR calculated for such SNPs would be infinite. However, power is low to detect such variants because they are typically at low frequency, and single-marker tests are underpowered to detect such variants [1]. Recent population growth increases the number of such mutations (**Fig. S2.4C**). When a mutation’s effect on fitness is independent of its effect on the trait (τ = 0), power is highest for SNPs with ORs between 1.5 and 2. SNPs with higher ORs are typically at low frequency in the population, reducing the power to detect them (**Fig. S2.4B**). Though the effect size on the liability scale in the population is not correlated with allele frequency (**Fig. S10B**), low-frequency SNPs tend to have larger ORs, simply because they are more likely to show larger relative differences in frequency between cases and controls (**Fig. S10D**). Again, population history has little effect on power (**Fig. S2.4B**). Recent growth increases the number of mutations that are present only in cases and that appear to have ORs of infinity (**Fig. S2.4D**). Growth also increases the number of mutations present only in controls that have ORs of 0. However, the median OR is still >1, reflecting the fact that cases carry more variants that are not carried by controls (rather than vice versa). This is expected as causal mutations are expected to increase risk of disease and as such, cases are expected to carry more of them.

**Figure S2.4: Properties of single-marker association tests for causal variants in a study of 1000 cases and 1000 controls, binned by the estimated odds ratio (*x*-axis).** Statistics were calculated for each simulation replicate and were averaged over the 1000 simulation replicates. An odds ratio of “Inf” refers to those variants that were found only in cases. **(A, C)** A SNP’s effect on the trait is correlated with its effect on fitness (τ = 0.5). (**B, D**) A SNP’s effect on the trait is independent of its effect on fitness (τ = 0). **(A, B)** Power. **(C, D)** Number of casual variants at different allele frequencies. Note that recent growth drastically increases the number of causal variants found exclusively in cases (green lines; BN+growth).

**REFERENCES**

1.     Kryukov GV, Shpunt A, Stamatoyannopoulos JA, Sunyaev SR. (2009) Power of deep, all-exon resequencing for discovery of human trait genes. Proc Natl Acad Sci U S A 106: 3871-3876.
